# Effect of intraoperative muscle relaxation reversal on the success rate of motor evoked potential recording in patients undergoing spinal surgery: a randomized controlled trial

**DOI:** 10.1186/s12871-023-02211-z

**Published:** 2023-08-25

**Authors:** Minyu Jian, Haiyang Liu, Fa Liang, Bo Ma, Lianjie Wang, Yang Zhou, Hui Qiao, Ruquan Han, Chengwei Wang

**Affiliations:** 1 Department of Anesthesiology, Beijing Tiantan Hospital, Capital Medical University, No. 119, Southwest 4th Ring Road, Fengtai District, Beijing, 100070 PR China; 2 Department of Anesthesiology, Beijing Fangshan Liangxiang Hospital, Beijing, China; 3 Department of Electrophysiology, Beijing Neurosurgical Institute, Beijing, China

**Keywords:** Muscle relaxation reversal, Motor evoked potentials, Spinal surgery

## Abstract

**Background:**

Partial neuromuscular blockade (NMB) has been applied for some surgeries to reduce bleeding and prevent patient movement for spinal surgery. Sugammadex selectively binds to rocuronium in the plasma and consequently lowers the rocuronium concentration at the neuromuscular junction. In this study, we aimed to observe whether the success rate of transcranial motor-evoked potential (TceMEP) can be increased by sugammadex compared with partial NMB during spinal surgery.

**Methods:**

Patients who underwent elective spinal surgery with TceMEP monitoring were randomly assigned to the sugammadex group and control group. Rocuronium was continuously infused to maintain the train of four counts (TOFc) = 2. The sugammadex group discontinued rocuronium infusion at the time of TceMEP monitoring and was infused with 2 mg/kg sugammadex; the control group was infused with the same dose of saline.

**Results:**

A total of 171 patients were included. The success rate of TceMEP monitoring in the sugammadex group was significantly higher than that in the control group. TceMEP amplitudes were greater in the sugammadex group than in the control group at 5 min, 10 min, and 20 min after the start of motor-evoked potential monitoring. The latencies of upper extremity TceMEPs monitoring showed no difference between groups. TOF ratios were greater in the sugammadex group at 5 min, 10 min, and 20 min after the start of motor-evoked potential monitoring. There were no adverse effects caused by sugammadex.

**Conclusions:**

Sugammadex can improve the success rate of motor-evoked potential monitoring compared with moderate neuromuscular blockade induced by continuous infusion of rocuronium in spinal surgery.

**Trial registration:**

The study was registered on clinicaltrials.gov.cn on 29/10/2020 (trial registration number: NCT04608682).

## Background

Intraoperative neuromonitoring (IOM) is frequently used during spinal surgeries that carry the risk of neurological damage. Real-time detection of compromised nerve function by IOM gives the surgeon an opportunity to address possible causes and avoid permanent injury. Transcranial motor evoked potentials (TceMEPs) are muscle action potentials elicited by transcranial brain stimulation and have been the most widely used method of IOM because of their high sensitivity for detecting neurologic injury. Electrical stimulation applied over the motor cortex allows compound motor action potentials to be recorded peripherally [1]. TceMEP monitoring plays an important role in preventing motor dysfunction during spinal surgery.

Neuromuscular blockade (NMB) acts at the neuromuscular junction and leads to a dramatic loss of TceMEP signals. For most cases requiring TceMEPs, the use of NMB is omitted after intubation [2, 3]. However, evidence is still conflicting regarding the optimal level of NMB during such monitoring since appropriate muscle relaxation facilitates surgery, optimizes anesthetic management, and prevents patient movement. Partial NMB (pNMB) induced by constant infusion of muscle relaxants according to the amplitude of muscle responses to “train of four” TOF has been applied in TceMEP monitoring [4–6]. Notably, this approach increases technical and interpretive complexity and runs the risk of inadvertently disabling TceMEPs at a critical moment. The incidence of monitoring failure and false-positive results was increased under pNMB [7, 8]. Sugammadex is a novel reversal agent that selectively binds to rocuronium in the plasma and consequently lowers the rocuronium concentration at the neuromuscular junction [9]. Multiple studies have confirmed the efficacy of reversing various levels of rocuronium block by Sugammadex [10–14]. Our previous study revealed that sugammadex could be used to enhance TceMEP waveform monitoring during spinal surgery requiring muscle relaxation [15]. We further evaluated the success rate of TceMEP recording under partial NMB and no NMB reversed by sugammadex in this randomized controlled study. We hypothesized that the muscle relaxation reversal effect of sugammadex can increase the success rate of TceMEP recording in spinal surgery.

## Methods

This prospective, single-center, parallel-group, assessor-blinded, randomized controlled trial was conducted in Beijing Tiantan Hospital, Capital Medical University, from August 16, 2021 to August 30, 2022 and was approved by the Institutional Review Board of Beijing Tiantan Hospital at May, 16 2021 (KY2021-082-02). The study was registered on clinicaltrials.gov.cn on 29/10/2020 (trial registration number: NCT04608682). Before randomization, written informed consent was obtained from all participants during preoperative evaluation. The trial protocol has already been published [15]. The study followed the Consolidated Standards of Reporting Trials (CONSORT) reporting guidelines. All methods were carried out in accordance with declaration of Helsinki.

Eligible candidates were patients from 18 to 65 years old undergoing thoracic or lumbar spinal surgery with TceMEPs monitoring and American Society of Anesthesiologists (ASA) physical status I to II. The exclusion criteria included BMI ≥ 35 kg/m^2^, history of epilepsy or use of antiepileptic drugs, neuromuscular disorder(s), personal history or family history of malignant hyperthermia, allergies to sugammadex, NMBs or other medication(s) used during general anesthesia, hemoglobin < 110 g/L, TceMEPs stimulation or recorded site infection, preoperative neurological dysfunction in both upper extremities, cardiac pacemaker, pregnancy and lactation, any other investigational drugs within 30 days of randomization or participation in another clinical trial within 30 days.

### Randomization and blinding

Enrolled participants were randomly allocated in a 1:1 ratio to the sugammadex group or the control group. A designated staff member who was not involved in anesthesia management or follow-up performed recruitment and generated the allocation randomization sequence. Randomization was performed by a computer-generated table. The allocation sequence was implemented through opaque, sealed, and stapled envelopes. Since the intervention included TOF monitoring, which was performed by anesthesiologists, the specific grouping information was not blinded to them, but the neurophysiologists, neurosurgeons, and the follow-up assessor were blinded to the grouping.

### Procedures and intervention

We used standard anesthesia management during TceMEP monitoring as described previously [16]. No premedication was administered. Anesthesia induction and maintenance were conducted with total intravenous anesthesia (TIVA) by a target-controlled infusion device (Marsh model, Master TCI-Diprifusor, Fresenius, Brezins, France). A propofol target concentration of 6 µg/mL and a remifentanil target concentration of 4 ng/mL were set for induction. Rocuronium (0.6 mg/kg) was used to achieve muscle relaxation. Tracheal intubation was performed after the patient failed to register signals using TOF. The infusion of propofol was adjusted to maintain a BIS (BIS Vista monitor, Aspect Medical Systems, Natick, MA) value of 40 to 50. More specific anesthesia management information, such as oxygen saturation, carbon dioxide saturation, and mechanical ventilation parameter settings, can be found in the published protocol [16].

Neuromuscular monitoring was achieved by ulnar nerve stimulation using a closed-loop muscle relaxant infusion system (CLMRIS-I, Guangxi VERYARK Technology Co., China.). All patients received a rocuronium infusion producing moderate blockade (at least two twitches in TOF) by the infusion system. The maintenance rate started from 0.6 µg/kg/min and was subsequently adjusted up to 12 µg/kg/min, and the bolus rate was 30 µg/kg/min. Rocuronium infusion was discontinued, and a bolus of sugammadex (2 mg/kg) was given while performing TceMEPs in the sugammadex group. The same volume of saline was administered to the control group while performing TceMEPs.

### Acquisition of TceMEPs

The acquisition of TceMEPs (Nicolet Neurological Workstation, WI) has been described previously [17]. Patients in both groups were monitored with TceMEPs. Recordings measured from the upper extremity abductor pollicis brevis muscles were conducted to avoid the interference of surgical manipulation on thoracic or lumbar levels for lower limb muscles. Thirty minutes after induction of anesthesia (T_B_), constant voltage stimulation began at 100 V to obtain the TceMEP threshold voltage. The stimulus intensity increased in steps of 20 V until the amplitudes (peak to peak) of TceMEPs > 50 µV were obtained. These voltage levels were considered TceMEP threshold intensities for monitoring in surgery. The TceMEP recording started at the time of dura opening or as surgeons’ request (T_0_). The neurophysiologists collected TceMEP waveforms twice under the same stimulation threshold if both waveforms were more than 50 µV, which was defined as a “repeatable” waveform. The success of TceMEPs was defined as collecting repeatable and stable TceMEPs waveforms (wave amplitude ≥ 50 µV) examined by neurophysiologists who were blinded to the grouping. The latencies (duration between the starting point of stimulation to the peak of the first negative wave) and amplitudes of TceMEPs in the upper extremities were recorded at 5 (T_5_), 10 (T_10_), 20 (T_20_), 30 (T_30_) and 60 (T_60_) minutes after the first TceMEP was performed.

### Outcome measures

The primary endpoint was the success rate of TceMEPs recording in the abductor pollicis brevis muscles of upper extremities 5 min after the first performing of TceMEPs (since the median time for reversal of moderate blockade caused by rocuronium with sugammadex is reported to be 1.3–1.7 min) [18]. The secondary endpoints included the mean value of amplitudes of TceMEPs in the abductor pollicis brevis muscles of both upper extremities at 5 (T_5_), 10 (T_10_), 20 (T_20_), 30 (T_30_) and 60 (T_60_) minutes after first performing TceMEPs, the mean value of latencies of TceMEPs in the abductor pollicis brevis muscles of both upper extremities at 5 (T_5_), 10 (T_10_), 20 (T_20_), 30 (T_30_) and 60 (T_60_) minutes after first performing TceMEPs, the thresholds that are required to obtain a dependable TceMEPs response, peak respiratory pressures, adverse effects of sugammadex, incidence of body movement, and recurrence of neuromuscular blockade. The incidence of body movement was classified as either nociception-induced movement (defined as “coughing” or reflexive limb movement temporally related to MEP stimulation) or excessive field movement (defined as grossly visible movement as determined by surgical and anesthesia teams).

### Statistical analysis

According to a previous study [19], we assumed that the success rate of TceMEPs was approximately 80% under pNMB and that the success rate of recordable TceMEPs was 95% after muscle relaxant reversal by sugammadex. Each group required 81 patients to achieve a power of 80% at a two-tailed significance level of 0.05, with a drop-out rate of 10%. We reported statistics with means and SDs or medians and IQRs as appropriate. The data were analyzed on an intention-to-treat basis.

The mean values of the amplitudes and latencies measured at different time points were analyzed by independent sample t tests. Repeated-measures ANOVA was used to check within-group differences at different time points. For categorical variables such as the incidence of adverse effects and body movement, the chi-square test or Fisher’s exact test was performed. We conducted all statistical analyses with SPSS version 26.0 (IBM Corporation, USA).

## Results

### Patients and baseline characteristics

**Fig. 1:**
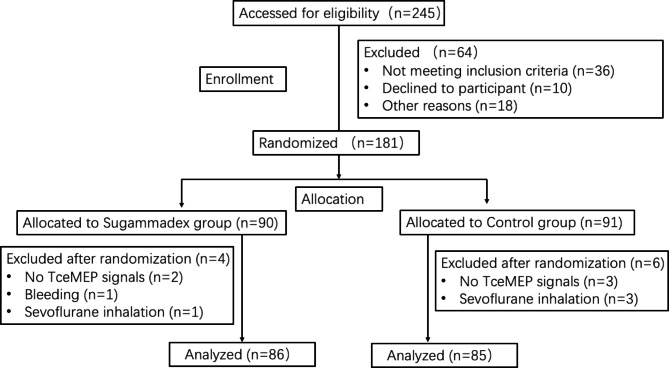
CONSORT flow diagram. TceMEP, transcranial electrical motor evoked potential

Of 245 patients who underwent thoracic or lumbar spinal surgeries from August 16, 2021, to August 30, 2022, 181 were recruited and randomly assigned to the sugammadex group (n = 90) or control group (n = 91). 10 patients were excluded after randomization for couldn’t detect TceMEP signals, bleeding, or sevoflurane inhalation during surgery (Fig. 1). 171 patients were analyzed. The 2 groups were balanced in baseline characteristics and preoperative data (Table 1).

**Table 1:** Baseline characteristics and preoperative data of the study population

	Sugammadex group N=86	Control group N=85	*P*	
Male/Female	43/43	43/42	>0.99	
Age(years)	51±12	48±14	0.29	
Height(cm)	165.3±7.8	166.2±7.1	0.41	
Weight(kg)	67.8±11.5	68.3±11.2	0.73	
Hemoglobin(g/L)	145.4±13.7	142.6±21.6	0.32	
Glucose(mmol/L)	5.74±1.7	5.89±0.8	0.11	
ASA I/II	7/79	11/74	0.33	
Diagnosis				
Thoracic	24	29	0.41	
Meningioma	8	11	0.47	
Ependymoma	3	2	0.99	
Gliomas	2	3	0.68	
Teratoma	1	0	0.99	
Neurofibroma	6	9	0.43	
Hemangioblastoma	2	1	0.99	
Thoracic spinal stenosis	2	3	0.68	
Lumbar	62	56	0.41	
Meningioma	11	13	0.66	
Ependymoma	8	7	0.99	
Gliomas	5	6	0.99	
Neurofibroma	9	7	0.79	
Hemangioblastoma	1	2	0.62	
Tethered cord	6	4	0.74	
Lipoma	2	1	0.99	
Lumbar disk herniation	5	4	0.99	
Lumbar spinal stenosis	15	12	0.68	
Blood loss(mL)	236±36	280±39	0.13	
Fluid infusion volume(mL)	2386±154	2381±142	0.68	
Operation time(hours)	4.4±1.7	4.1±1.4	0.16	

Data are presented as mean ± SD or n. Abbreviations: ASA: American society of Aneshesiologists (ASA) physical status classification system

### Outcome

**Fig. 2:**
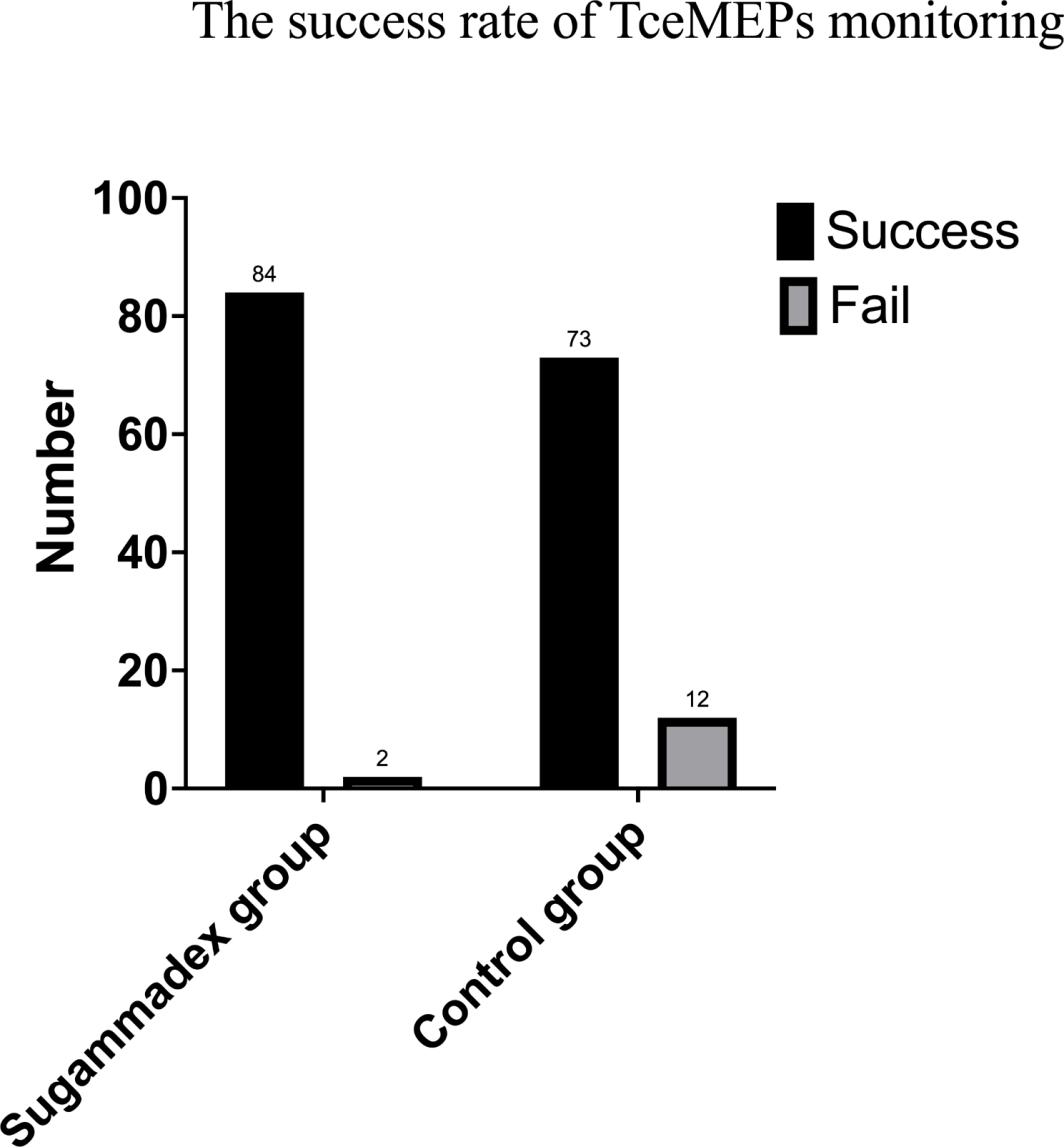
The success rate of upper extremity abductor pollicis brevis muscles transcranial motor evoked potentials (TceMEPs) in both groups, the success rate in the sugammadex group was significantly higher than control group (97.7% vs. 85.9%, *P* = 0.0049)

The success rate of TceMEP monitoring in the sugammadex group was significantly higher than that in the control group (97.7% vs. 85.9%, *P* = 0.0049) (Fig. 2).

**Fig. 3:**
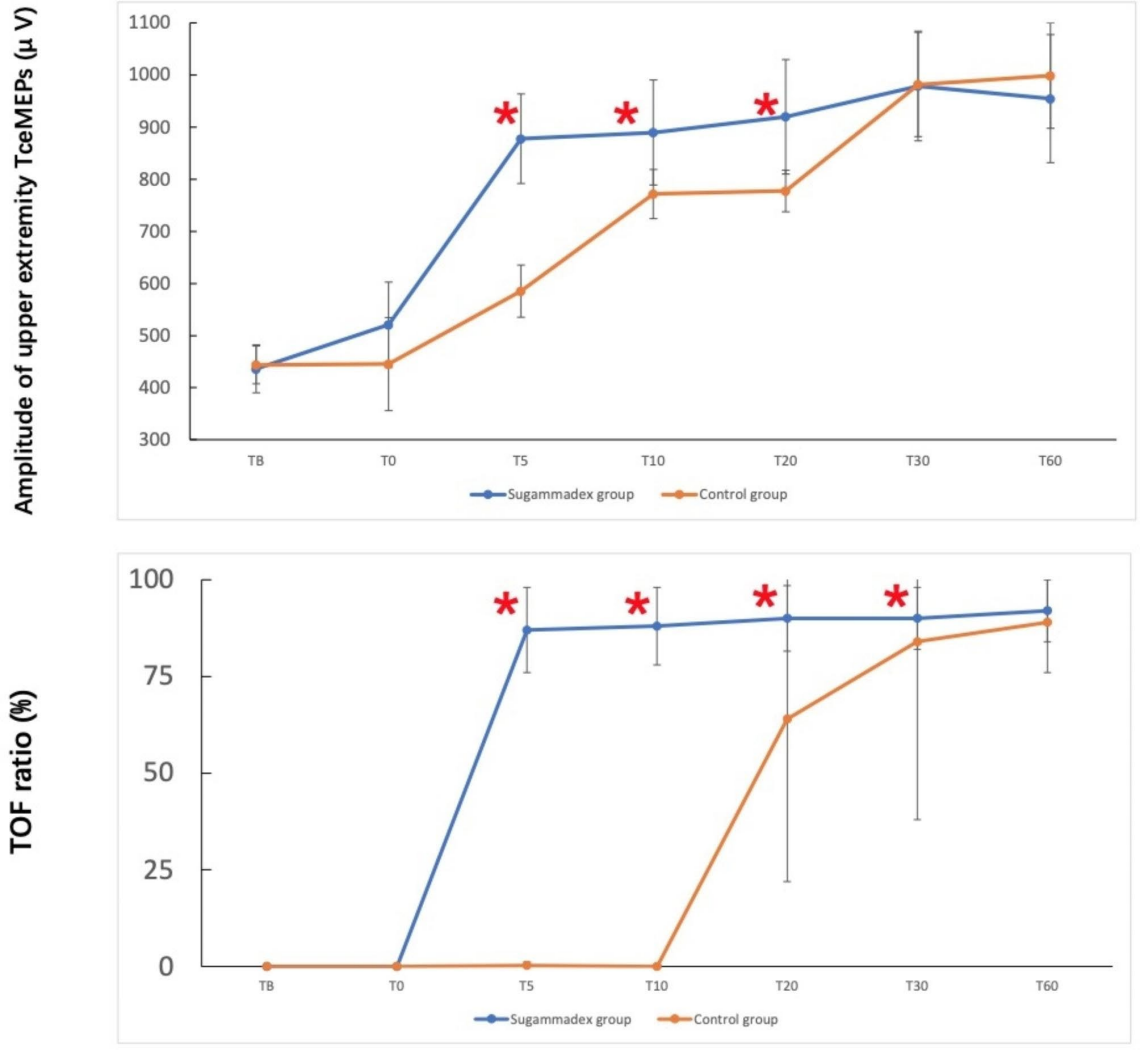
Upper: The amplitudes of upper extremity abductor pollicis brevis muscles transcranial motor evoked potentials (TceMEPs) in both groups. TceMEP amplitudes were significantly greater in the sugammadex group than in the control group at T_5_ (*P* = 0.01), T_10_ (*P* = 0.02), and T_20_ (*P* = 0.02). There was no difference at T_B_ (*P* = 0.61), T_30_ (*P* = 0.55) or T_60_ (*P* = 0.17). Lower: The TOF ratios in both groups. The TOF ratios were significantly greater in the sugammadex group than in the control group at T_5_ (*P* < 0.0001), T_10_ (*P* < 0.0001), T_20_ (*P* = 0.0001) and T_30_ (*P* = 0.0024). There was no difference at T_B_ (*P* = 0.12) or T_60_ (*P* = 0.13). * indicates *P* < 0.05

The latencies of upper extremity TceMEPs recording showed no difference between groups at baseline (T_B_) (*P* = 0.23), the time of TceMEPs recording (T_0_) (*P* = 0.51), 5 min (T_5_) (*P* = 0.97), 10 min (T_10_) (*P* = 0.78), 20 min (T_20_) (*P* = 0.93), 30 min (T_30_) (*P* = 0.87), and 60 min (T_60_) after TceMEPs recording (*P* = 0.86). TceMEP recording amplitudes were significantly greater in the sugammadex group than in the control group at T_5_ (*P* = 0.01), T_10_ (*P* = 0.02), and T_20_ (*P* = 0.02). There was no difference at T_B_ (*P* = 0.61), T_30_ (*P* = 0.55) or T_60_ (*P* = 0.17). The amplitudes were consistent with the TOF ratios, which were significantly greater in the sugammadex group than in the control group at T_5_ (*P* < 0.0001), T_10_ (*P* < 0.0001), T_20_ (*P* = 0.0001) and T_30_ (*P* = 0.0024). There was no difference at T_B_ (*P* = 0.12) or T_60_ (*P* = 0.13) (Fig. 3). The stimulus intensity was significantly lower in the sugammadex group than in the control group (140 (140,140) vs. 180 (143,200), *P* < 0.001).

MAP, HR, PETCO_2_, BIS, body temperature and peak airway pressures showed no difference between the two groups at all timepoints (Table 2). We didn’t observe any intraoperative movement in both groups. There were no adverse effects caused by sugammadex.

**Table 2:** Perioperative physiological characteristics of the study

		T_B_	T_0_	T_5_	T_10_	T_20_	T_30_	T_60_
MAP (mmHg)	Sugammadex	100±15	88±10	87±10	87±9	87±10	87±10	85±13
	Control	97±10	86±10	85±10	84±9	85±10	85±11	86±10
	*P*	0.05	0.11	0.08	0.13	0.09	0.29	0.46
h (bpm)	Sugammadex	72±13	62±10	64±12	58±10	58±10	58±9	59±10
	Control	74±10	65±11	64±11	60±9	60±10	60±9	60±10
	*P*	0.27	0.08	0.87	0.15	0.18	0.08	0.38
PETCO_2_ (mmHg)	Sugammadex	/	29±3	29±3	29±3	28±3	29±3	29±3
	Control	/	29±3	30±3	29±3	29±3	29±3	30±3
	*P*	/	0.15	0.06	0.31	0.32	0.08	0.10
BIS	Sugammadex	92±5	43±4	43±5	43±4	44±4	43±4	44±6
	Control	93±4	43±7	42±5	44±6	42±6	43±5	44±6
	*P*	0.10	0.99	0.73	0.48	0.15	0.86	0.70
Body temperature (°C)	Sugammadex	36.6±0.3	36.0±0.4	35.9±0.3	36.7±0.3	36.3±0.5	36.5±0.4	36.6±0.3
	Control	36.6±0.2	36.3±0.5	36.3±0.4	36.7±0.3	36.2±0.2	36.3±0.4	36.6±0.5
	*P*	0.18	0.41	0.32	0.89	0.64	0.15	0.33
Peak airway pressures (mmHg)	Sugammadex	/	17±3	17±3	28±3	17±3	17±3	17±3
	Control	/	17±3	17±3	29±3	17±3	16±3	17±3
	*P*	/	0.44	0.41	0.06	0.45	0.19	0.84

Data are presented as mean ± SD. Abbreviations: MAP: mean artery pressure, HR: heart rate, PETCO_2_: partial pressure of end-tidal carbon dioxide, BIS: Bispectral Index

## Discussion

TceMEP monitoring during spinal surgery can assess the functional integrity of motor pathways to early detect motor dysfunction and allow intervention before damage becomes irreversible [20]. pNMB can optimize the surgical field, prevent unacceptable movements or coughs, and reduce bleeding during surgery, thus some surgical procedures may prefer pNMB over no NMB [21]. Our study evaluated the success rate of TceMEP monitoring in patients undergoing spinal surgery on intraoperative reversal of muscle relaxants. Sugammadex can improve the success rate of TceMEP monitoring compared with moderate NMB induced by continuous infusion of rocuronium in spinal surgery.

MEPs directly monitor the anterolateral columns and are helpful in detecting and preventing neurological injuries caused by anterior compression or by impaired blood flow to the anterior spinal cord. Changes in anesthetic management lead to changes in MEPs. Comparing with inhalational anesthetics, the benefits of intravenous agents for MEP monitoring are less interference with alpha motor neuron excitability [22, 23]. Therefore, our study applies standard anesthetic regimens by total intravenous anesthesia with propofol and remifentanil to obtain an accurate recording. Both groups experienced similar BIS values and optimal physiological variables in our study, which are crucial to detect TceMEP signals.

pNMB induced by continuously infusing a low-dose muscle relaxant can be used in spinal surgery requiring TceMEPs, and those who advocate for pNMB may be concerned about the complete omission of muscle relaxant resulting in difficulty in exposing the surgical field, and the risk of unexpected patient movement [24]. However, a major disadvantage of this technique is that it requires complicated anesthetic management including strict control of the muscle relaxant based on neuromuscular monitoring [25]. Our study maintained a constant moderate muscle relaxation by a closed-loop continuous infusion system. Another shortcoming is that patients with pNMB generate smaller TceMEP amplitudes than patients without muscle relaxant. Therefore, a higher stimulation intensity is necessary for partially paralyzed patients. The stimulation intensity was significantly lower in the sugammadex group than in the control group (140 (140,140) vs. 180 (143,200), *P* < 0.001) in our study. High stimulus intensity can activate the deep subcortical motor pathways and bypass higher cortical levels, which might generate MEP signals from the deepening of the contralateral limbs despite cortical ischemia. In that case, the incidence of monitoring failure and false positives will be increased [4].

Sugammadex can quickly and safely reverse neuromuscular blockade by encapsulating rocuronium and vecuronium, enabling complete recovery of neuromuscular function. Previous studies have explored the effect of sugammadex on the amplitudes of TceMEP monitoring. Venkatraghavan et al. [26] demonstrated a 200% increase in the MEP amplitude in the first dorsal interosseous muscle at 3 min following reversal of neuromuscular blockade with sugammadex (2 mg/kg) in patients with cervical myelopathy. Similarly, Liu et al. [15] showed that sugammadex (2 mg/kg) improved the amplitudes of upper extremity TceMEP monitoring signals in 5 min. Our study focused on the success rate of TceMEP recording under pNMB. The success of TceMEPs was defined as collecting repeatable and stable TceMEPs waveforms, which indicates the feasibility of TceMEP recording, is of importance during the clinical setting. Otherwise, the accumulated dose of rocurinum is obviously higher under these circumstances; therefore, the onset time and dosage of sugammadex need to be confirmed. The success rate of TceMEPs was significantly higher after reversal of sugammadex, as was the TOF values in our study.

Previous study has revealed sugammadex can cause cortical arousal and increased the BIS values [27], the possible mechanism is that muscle activity led to afferent input to brain arousal centers. NMB has a sedative effect by decreasing muscle activity [28, 29]. In our study, we did not notice any BIS value changes after administration of sugammadex, and there were no other clinical signs of patient arousal, e.g., significant increases in hemodynamic parameters. Therefore, we believe that the increase in the success rate in TceMEP signals with sugammadex is mainly due to the reversal of residual NMB [29].

There are some limitations to our study. We chose TceMEPs signals from upper extremities during surgical procedures on the thoracic or lumbar spine in our study, allowing the neurophysiological team to isolate changes that occur only in the lower extremities (which are a result of surgical technique) from changes in the upper extremity signals (which may be caused by anesthetic managements) to avoid the interference of surgery manipulation. This may limit the generalization of our data to other muscle groups, especially from the lower extremities, due to the difference in the recovery rate of each muscle. Second, some concerns may be raised about unexpected patient movement that develops after the administration of sugammadex [26], and neuromuscular blockade may need to be re-established. We did not observe any body movement in our study after sugammadex, and further studies may need to confirm the safety of the drug. Third, we did not evaluate the correlation between TceMEPs and postoperative motor function in either group.

## Conclusions

Sugammadex can improve the success rate of motor-evoked potential monitoring compared with moderate neuromuscular blockade induced by continuous infusion of rocuronium in spinal surgery. Our findings highlight the role of sugammadex in reversing neuromuscular blockade during motor-evoked potential monitoring.

## Data Availability

All data generated or analysed during this study are included in this published article.

## Declarations

### Patient and Public Involvement

Patients and the public were not involved in the trial design. Participants will have access to the findings of the study on request.
